# Asian Swamp eel *Monopterus albus* Population Structure and Genetic Diversity in China

**DOI:** 10.3389/fgene.2022.898958

**Published:** 2022-05-27

**Authors:** Weiwei Lv, Quan Yuan, Weiwei Huang, Xiaolin Sun, Weiguang Lv, Wenzong Zhou

**Affiliations:** Eco-Environmental Protection Research Institute, Shanghai Academy of Agricultural Sciences, Shanghai, China

**Keywords:** *Monopterus albus*, population structure, genetic diversity, snps, China

## Abstract

The Asian swamp eel (*Monopterus albus*) is one of the most widely distributed freshwater fish in China. In this study, we identified the single nucleotide polymorphisms (SNPs) of *M. albus* from 19 wild populations in China using restriction-site associated DNA sequencing (RAD-seq), and used SNP markers to investigate the swamp eel the genetic diversity and population genetic structure. A total of 8941794 SNPs were identified. Phylogenetic and principal component analysis suggested that the 19 populations were clustered into four groups: The Jiaoling County (JL) and Poyang Lake (PYH)populations in Group Ⅰ; the Chengdu City (CD), Dali City (YN), Eli Village (EL), Dongting Lake (DTH), Huoqiu County (HQ), and Chaohu Lake (CH) populations in Group Ⅱ; the Puyang City (PY), Chongming Island (CM), Tai Lake (TH), Gaoyou Lake (GYH), Weishan Lake (WSH), Haimen City (HM), Hongze Lake (HZH), Baiyangdian Lake (BYD), Dagushan (DGS), and Pinghu City (PH) populations in group Ⅲ; and the Lingshui County (LS) populations in Group Ⅳ. All 19 populations may have evolved from four ancestors. The genetic diversity was relatively high in CM, GYH, and HM; and low in LS, EL, and JL. The LS, and CM populations had the highest and lowest differentiation from the other populations, respectively. These findings provide new insights for germplasm resources protection and artificial breeding of *M. albus*.

## 1 Introduction

Asian swamp eel (*Monopterus albus*), which is commonly found in rice fields, muddy ponds, and swamp areas (Ip et al., 2004; Siang et al., 2007), is distributed widely in the Indo-Malayan Archipelago, Japan, Asiatic Russia, China, Burma, and northern India (Supiwong et al., 2019). In recent years, due to the increasing use of various agricultural chemicals and the discharge of industrial pollutants, the habitat of wild *M. albus* has deteriorated and the germplasm resources of *M. albus* have been greatly reduced by fishing. In fact, less the 0.01% of available aquatic habitat comprise freshwater habitats; however, they are home to almost 50% of all 34,000 fish species. Thus, the complex environment of freshwater fish is a good model to study speciation events and genetic mutation (Bloom et al., 2013). However, the fragmented and isolated freshwater environments cause allopatric speciation events, and several examples of sympatric speciation have been reported (Bolnick and Fitzpatrick, 2007; Seehausen and Wagner, 2014). The eel population in China has been differentiated into local populations with different genetics, which will inevitably lead to the mutual penetration of species genes and cause irreparable damage to the quality of germplasm resources.

Research into germplasms has improved our understanding of recent speciation events. In China, Cai et al. (2012) investigated the phylogeny of the mitochondrial control region of 167 swamp eels representing 12 regional populations, and these *M. albus* could be classified into five genetic lineages. Chen et al. sequenced the mitochondrial genome of rice field *M. albus* populations from four different areas, which classified them into two groups: Those from Guangxi province and the other population (Chen et al., 2016). Liang et al. used mitochondrial DNA to investigate swamp eel genetic variation in populations from six dominant farming areas, and revealed marked genetic differentiation among the six populations (Liang et al., 2016). In Indonesia, molecular genetic data was used to investigate the swamp eel’s phylogeny and taxonomy, which revealed two distinct Indonesian forms of *M. albus*, one that was indigenous to Indonesia and one that was distributed widely in Indonesia and in other Southeast Asian countries lying to the north. According to the phylogenetic analysis, the southern forms may have evolved more recently from the older lineages located in northern Asia (Arisuryanti, 2016). In Matsumoto’s study, the molecular phylogeny of mitochondrial 16 s rRNA sequences was investigated using 84 swamp eel samples from 13 locations in East and Southeast Asia, which delineated the eels genetically into three clades (Southeast Asia, Ryukyu Islands, and China–Japan) based on geographical populations (Matsumoto et al., 2010). Therefore, analyses of germplasm resources analysis provides the basis to understanding *M. albus* biogeography and evolutionary diversification, thus contributing to our knowledge regarding *M. albus* taxonomy *via* cryptic species discovery.

The swamp eel is one of the most popular freshwater aquaculture fish for human consumption, yielding 320,966 tons in 2012 (Liang et al., 2016). However, this yield is not sufficient to satisfy consumer demands (Sun et al., 2017). Therefore, it is imperative to study the genetic diversity of *M. albus*, which plays a positive role in the conservation of wild *M. albus* resources and germplasm breeding. Single Nucleotide Polymorphisms (SNPs) are a highly popular type of marker (Vignal et al., 2002). SNPs require assessment of neutrality, are appropriate for use in population genetics, and have higher stability, more polymorphisms, and are easier to detect than microsatellite molecular markers. For genetic analyses, multi-allelic systems can be defined by reconstructing haplotypes, which overcomes the limitations resulting from SNPs’ low heterozygosity. Therefore, SNPs are more suitable for germplasm analysis (Coates et al., 2009; Hauser et al., 2011).

In this study, we identified SNP markers from 19 *M. albus* populations in China using restriction-site associated DNA sequencing (RAD-seq). Then, we analyzed their genetic differentiation, population genetic structure, and genetic diversity based on the SNP data, with the aim of accelerating the process of molecular breeding in swamp eels.

## 2 Materials and Methods

### 2.1 Sampling and DNA Extraction

**FIGURE 1:**
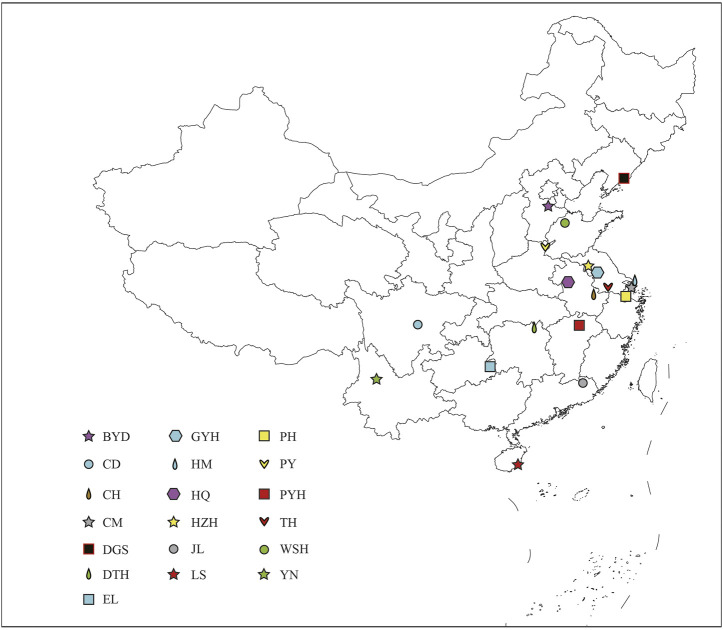
Sampling sites of 19 populations in China. The abbreviations represent the sampling sites: BYD, Baiyangdian Lake; CD, Chengdu City; CH, Chaohu Lake; CM, Chongming Island; DGS, Dagushan; DTH, Dongting Lake; EL, Eli village; GYH, Gaoyou Lake; HM, Haimen City; HQ, Huoqiu County; HZH, Hongze Lake; JL, Jiaoling County; LS, Lingshui County; PH, Pinghu City; PY, Puyang City; PYH, Poyang Lake; TH, Tai Lake; WSH, Weishan Lake; YN, Yunnan.

A total of 137 wild eels were collected from 19 sampling points in China (Figure 1; Supplementary Table S1). One Gram of muscle tissue of each healthy eel was harvested in liquid nitrogen. For RAD-seq analysis, we selected 5 to 10 individuals of different genders and growth stages at each site. The cetyltrimethyl ammonium bromide (CTAB) method was used extract total genomic DNA from the samples (Doyle and Doyle, 1990). The quality of the extracted DNA was assessed using 0.8% agarose gel electrophoresis, and the DNA concentration was quantified using an ultraviolet spectrophotometer (Thermo Fisher Scientific, Waltham, MA, United States ).

### 2.2 Library Preparation and Sequencing

Using the method detailed by Baird et al. (2008), 500 ng of DNA per sample were used to prepare the RAD libraries. Briefly, restriction enzyme EcoR I was used to digest the genomic DNA for 15 min at 37°C. The digested samples were ligated with the P1 Illumina adapter (Illumina Inc., San Diego, CA, United States ), pooled, and then sheared randomly using a Bioruptor (Diagenode, Liege, Belgium). A MinElute Gel Extraction Kit (Qiagen, Dusseldorf, Germany) was used to purify the resultant 300–500 bp DNA fragment. A Quick Blunting kit Enzyme Mix (NEB, Ipswich, MA, United States ) was employed to repair the dsDNA ends, followed by ligation of a modified Illumina P2 adapter. Finally, the DNA fragments were purified and quantified, followed by amplification using the PCR conditions comprising: 98°C for 2 min; 13 cycles at 98°C for 30 s, 60°C for 30 s and 72°C for 15 s; and a final extension at 72°C for 5 min. The Illumina Novaseq system at Personalbio Co. Ltd. (Shanghai, China) was used to sequence the DNA. All of the swamp eel sequencing data were submitted to the SRA database in NCBI under accession numbers SRX13783495-SRX13783631. The statistics of the raw data obtained were shown in Supplementary Table S2.

### 2.3 Molecular Marker Discovery

Initially, the Axe package (Murray and Borevitz, 2018) was used to assign the individual samples *via* their unique nucleotide barcodes. The read quality was examined using *Fastp* software (Chen et al., 2018). Any low quality reads, including reads with more than 40% nucleotides and those with a quality value lower than 20 (equal to a 1% sequencing error) were trimmed or discarded (Supplementary Table S3). Trimmed reads were mapped to the reference of *M. albus* (GenBank No: GCA_001952655.1, Supplementary Table S3) using *BWA* MEM software under default mapping parameters (Li and Durbin, 2010). We assigned molecular markers using the *GATK* pipeline, which incorporates insertion/deletion (InDel)-realignment and mark-duplication, and identifies variants across all samples simultaneously using the *HaplotypeCaller* program in *GATK 4.1* (McKenna et al., 2010). Variants were filtered using standard hard filtering parameters according to the *GATK* pipeline best practices. In detail, SNPs were obtained with a Mapping Quality ≥40. Lastly, variants with a two-fold sequencing depth and a call rate over 70% were used to construct linkage maps.

### 2.4 Phylogenetic and Principal Component Analysis

Based on the SNP sites, Phylogenetic was generated using the maximum Likelihood (ML) algorithm in the *FastTree* software (http://www.microbesonline.org/fasttree/). The ML bootstrap (MLBS) analyses were conducted using *FastTree* with 1,000 replicates under the GTR + CAT model. We also performed principal component analysis (PCA) based on SNP data from 137 individuals. This analysis was carried out in the *GCTA* software (Yang et al., 2011).

### 2.5 Population Genetic Structure

To cluster each ancient genome in the study populations, we used the unsupervised maximum-likelihood clustering algorithm in the ADMIXTURE package (Alexander et al., 2009). For the 137 eels, ADMIXTURE with default setting was run for initial clustering for k = 2 to k = 10 ancestral clusters. The optimal K value was determined by the coefficient of variation (CV) error values.

### 2.6 Population Genetic Statistics

The POPULATIONS program in the *STACKS* pipeline, with default parameters (Catchen et al., 2011; 2013), was used to calculate the Pairwise FST values and population genetic statistics, including observed heterozygosity (Hobs), Wright’s F-statistic (FIS), and nucleotide diversity (*π*).

## 3 Results

### 3.1 SNPs Analysis in the 19 Populations of Swamp eel

A total of 8941794 SNPs were detected in the 19 eel populations, and the number of SNPs in the four detected genotypes in each population are shown in Supplementary Table S4 (SNP data have been submitted to NCBI). Then, the distribution of SNPs in the scaffolds with the top 20 l was further determined (Supplementary Figure S1). The distribution of SNP mutations was not uniform (Supplementary Figure S1A). Mutation spectrum and base bias analysis showed that genome-wide SNP mutations could be divided into six types, with T:A > C:G and C:G > T:A as the main SNP mutants (Supplementary Figure. S1B). Further analysis showed no base preference before and after the SNP mutation sites (Supplementary Figure S1C). ANNOVAR was used to annotate population SNP loci (Supplementary Table S5), and the results showed that mutation sites were mainly concentrated in intronic and intergenic regions.

### 3.2 Phylogenetic and Principal Component Analysis

**FIGURE 2:**
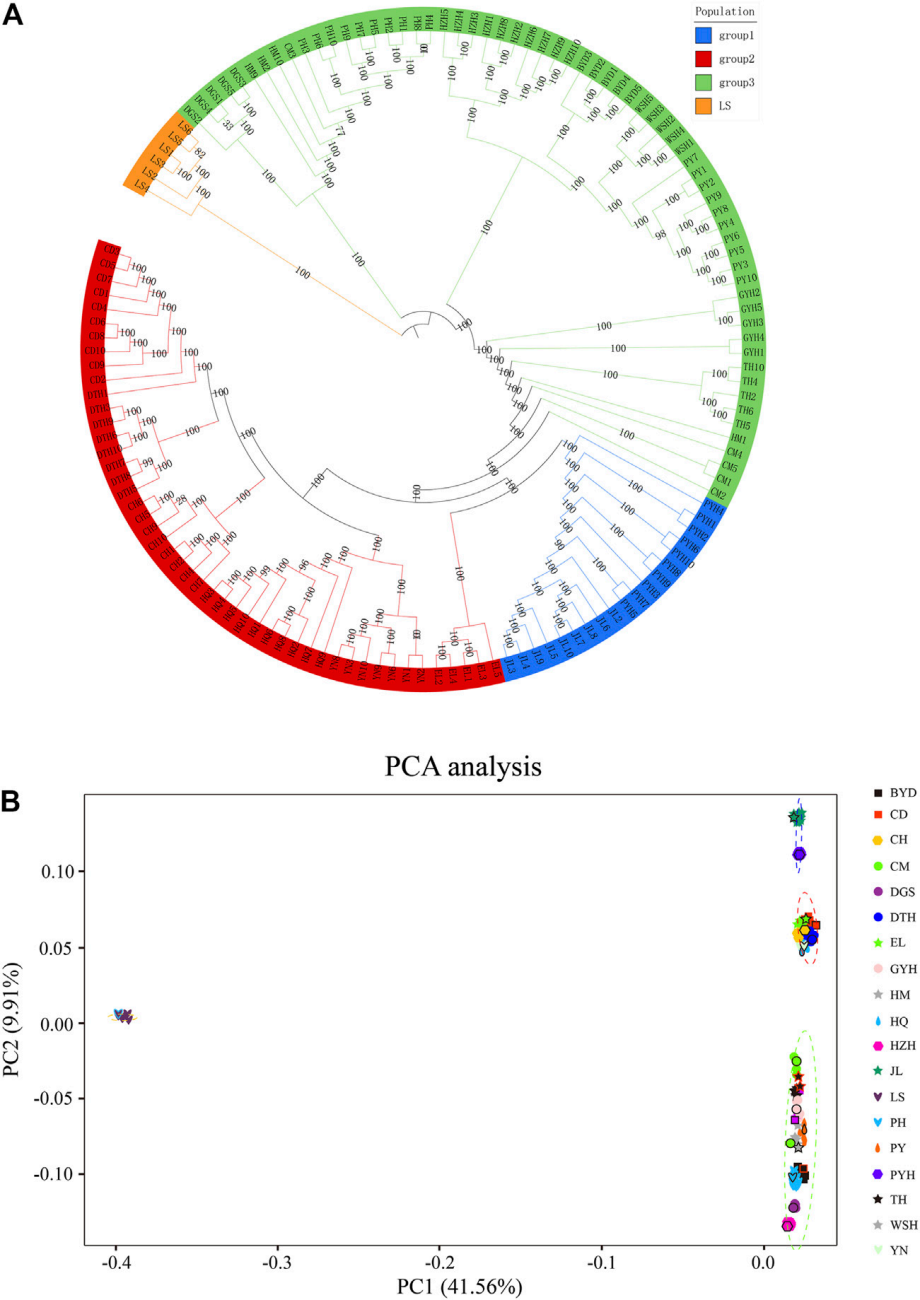
Phylogenetic tree **(A)** and principal component analysis (PCA) **(B)** based on single nucleotide polymorphisms (SNPs). The abbreviations represent sampling sites: BYD, Baiyangdian Lake; CD, Chengdu City; CH, Chaohu Lake; CM, Chongming Island; DGS, Dagushan; DTH, Dongting Lake; EL, Eli village; GYH, Gaoyou Lake; HM, Haimen City; HQ, Huoqiu County; HZH, Hongze Lake; JL, Jiaoling County; LS, Lingshui County; PH, Pinghu City; PY, Puyang City; PYH, Poyang Lake; TH, Tai Lake; WSH, Weishan Lake; YN, Yunnan.

Figure 2 shows the phylogenetic tree (Figure 2A) and PCA analysis (Figure 2B) constructed using genomic SNP markers. The phylogenetic tree suggested that the 19 populations of eels could be classified into four groups based on their genetic relationship distance. Group I comprised the Jiaoling County (JL) and Poyang Lake (PYH) populations; Group II comprised the Chengdu City (CD), Dali City (YN), Eli Village (EL), Dongting Lake (DTH), Huoqiu County (HQ), and Chaohu Lake (CH) populations; Group III comprised the Puyang City (PY), Chongming Island (CM), Tai Lake (TH), Gaoyou Lake (GYH), Weishan Lake (WSH), Haimen City (HM), Hongze Lake (HZH), Baiyangdian Lake (BYD), Dagushan (DGS), and Pinghu City (PH) populations; and Group Ⅳ comprised the Lingshui County (LS) population. The inferences from the phylogenetic tree were confirmed by the PCA analysis.

### 3.3 Genetic Structure of the Populations

**FIGURE 3:**
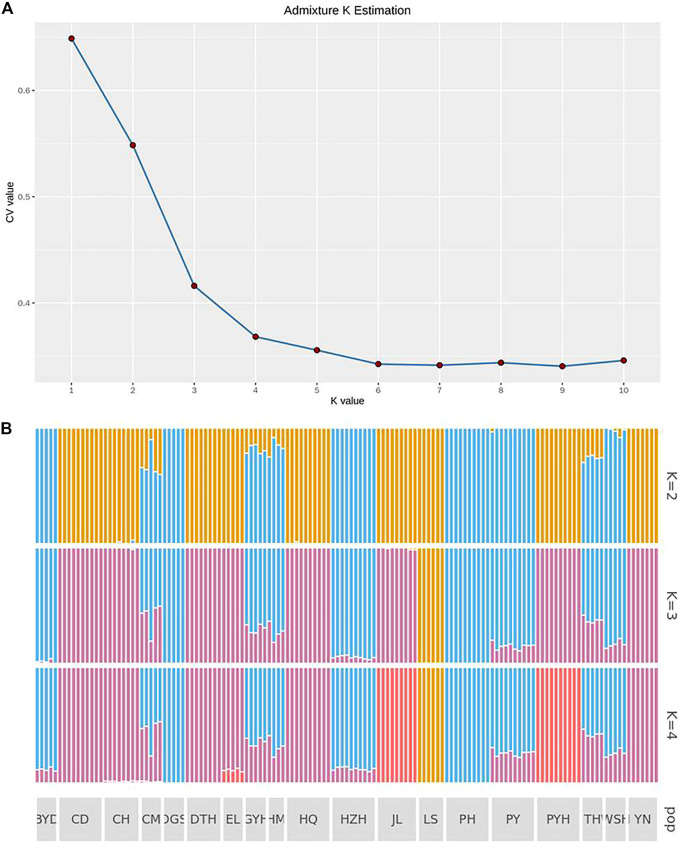
Analysis of population genetic structure **(A)** when the ancestor number is assumed to be 2–4 and the possible K value **(B)** by using admixture K estimation. The abbreviations represent the sampling sites: BYD, Baiyangdian Lake; CD, Chengdu City; CH, Chaohu Lake; CM, Chongming Island; DGS, Dagushan; DTH, Dongting Lake; EL, Eli village; GYH, Gaoyou Lake; HM, Haimen City; HQ, Huoqiu County; HZH, Hongze Lake; JL, Jiaoling County; LS, Lingshui County; PH, Pinghu City; PY, Puyang City; PYH, Poyang Lake; TH, Tai Lake; WSH, Weishan Lake; YN, Yunnan.

Figure 3A shows the CV errors under different K values calculated by ADMIXTURE software (http://dalexander.github.io/admixture). When the K value was 4, the population genetic structure was closest to the real situation. According to Figure 3B, the LS group evolved from a separate ancestor (ancestor 1). The CD, DTH, HQ and YN populations appeared to have evolved from common ancestor 2; DGS and PH evolved from common ancestor Ⅲ; and JL and PYH evolved from common ancestor 3. The BYD, GYH, HM, HZH, PY, and WSH populations may have been a heterozygous population, with crossing of ancestors 2 and 3. The CH and EL populations had genes from both ancestor 3 and 4. Notably, the CM population had the largest number of ancestors, being descended from three of them (2, 3, and 4).

### 3.4 Genetic Diversity Analysis

The genetic diversity indices of the 19 eel populations were shown in Table 1. We found that the observed heterozygosity of all populations, except for LS and CM, was lower than the expected heterozygosity. The nucleotide diversity (Pi) was relatively high in CM (0.19), GYH (0.17), and HM (0.17) and low in LS (0.01), EL (0.04), and JL (0.05). The inbreeding coefficient (Fis) was relatively high in HZH (0.14), TH (0.10), and WSH (0.10); and low in LS (0.003), EL (0.02), and JL (0.02).

**TABLE 1:** The genetic diversity of the *M. albus* in 19 populations based on the high consistency population single nucleotide polymorphisms (SNPs).

Pop ID	Num Indv	Obs Het	Obs Hom	Exp Het	Exp Hom	Pi	Fis
BYD	3.43	0.08	0.92	0.10	0.90	0.12	0.07
CH	4.72	0.08	0.92	0.10	0.90	0.11	0.07
CM	3.86	0.17	0.83	0.16	0.84	0.19	0.04
DGS	4.05	0.06	0.94	0.06	0.94	0.07	0.03
DTH	5.59	0.07	0.93	0.09	0.91	0.11	0.07
EL	3.81	0.03	0.97	0.04	0.96	0.04	0.02
GYH	3.48	0.13	0.87	0.14	0.86	0.17	0.08
JL	7.78	0.04	0.96	0.05	0.95	0.05	0.02
HM	3.16	0.13	0.87	0.14	0.86	0.17	0.08
LS	4.82	0.01	0.99	0.01	0.99	0.01	0.00
HQ	7.87	0.07	0.93	0.09	0.91	0.10	0.06
HZH	5.53	0.09	0.91	0.13	0.87	0.15	0.14
PH	7.84	0.10	0.90	0.13	0.87	0.14	0.08
PY	8.25	0.11	0.89	0.13	0.87	0.13	0.06
PYH	6.25	0.05	0.95	0.06	0.94	0.06	0.04
CD	8.84	0.07	0.93	0.08	0.92	0.09	0.06
TH	3.28	0.10	0.90	0.13	0.87	0.15	0.10
WSH	2.96	0.08	0.92	0.11	0.89	0.14	0.10
YN	5.58	0.08	0.92	0.09	0.91	0.10	0.04

The abbreviations in the PopID column represent the sampling sites; BYD, Baiyangdian Lake; CD, Chengdu City; CH, Chaohu Lake; CM, Chongming Island; DGS, Dagushan; DTH, Dongting Lake; EL, Eli village; GYH, Gaoyou Lake; HM, Haimen City; HQ, Huoqiu County; HZH, Hongze Lake; JL, Jiaoling County; LS, Lingshui County; PH, Pinghu City; PY, Puyang City; PYH, Poyang Lake; TH, Tai Lake; WSH, Weishan Lake; YN, Yunnan.

### 3.5 Population Differentiation

Table 2 showed the Fst values between different populations. The Fst values were greater than 0.15 in most populations, indicating significant genetic differentiation between the populations. The LS population had the highest degree of differentiation from other populations, in which the Fst value was 0.64–0.89. The Fst values between CM and 15 populations (except for DGS, JL, and LS populations) were lower than 0.25, indicating low differentiation degrees. The lowest Fst values were observed between DTH and CH (0.09) and between YN and HQ (0.09). The highest values of Fst were found between LS and EL (0.89).

**TABLE 2:** The F*st* distribution in 19 *M. albus* populations.

Fst	CH	CM	DGS	DTH	EL	GYH	JL	HM	LS	HQ	HZH	PH	PY	PYH	CD	TH	WSH	YN
BYD	0.29	0.19	0.34	0.29	0.43	0.19	0.45	0.21	0.77	0.29	0.17	0.23	0.15	0.40	0.33	0.22	0.19	0.32
CH		0.15	0.37	0.09	0.23	0.19	0.31	0.20	0.74	0.14	0.23	0.28	0.21	0.25	0.13	0.19	0.25	0.15
CM			0.25	0.15	0.23	0.12	0.29	0.11	0.64	0.19	0.14	0.16	0.16	0.24	0.19	0.12	0.16	0.19
DGS				0.37	0.54	0.27	0.54	0.28	0.84	0.39	0.24	0.25	0.27	0.49	0.40	0.30	0.34	0.41
DTH					0.23	0.19	0.31	0.20	0.74	0.15	0.23	0.29	0.22	0.26	0.12	0.19	0.26	0.15
EL						0.29	0.45	0.31	0.89	0.26	0.31	0.35	0.29	0.38	0.24	0.31	0.39	0.30
GYH							0.34	0.14	0.69	0.22	0.12	0.19	0.16	0.29	0.23	0.14	0.17	0.23
JL								0.34	0.86	0.34	0.36	0.39	0.35	0.18	0.35	0.35	0.41	0.36
HM									0.69	0.23	0.14	0.15	0.17	0.30	0.24	0.14	0.19	0.24
LS										0.74	0.67	0.67	0.68	0.83	0.76	0.71	0.75	0.77
HQ											0.25	0.31	0.22	0.28	0.19	0.22	0.25	0.09
HZH												0.17	0.15	0.31	0.27	0.15	0.15	0.26
PH													0.22	0.35	0.33	0.20	0.22	0.31
PY														0.31	0.26	0.17	0.12	0.23
PYH															0.29	0.30	0.37	0.30
CD																0.23	0.29	0.19
TH																	0.20	0.23
WSH																		0.27

Fst values between 0.15 and 0.25 represent a significant difference, Fst values > 0.25 represent an extremely significant difference, Fst = 0–0.05 indicates low genetic differentiation, and Fst = 0.05–0.15 indicates moderate genetic differentiation among populations. The abbreviations represent the sampling sites: BYD, Baiyangdian Lake; CD, Chengdu City; CH, Chaohu Lake; CM, Chongming Island; DGS, Dagushan; DTH, Dongting Lake; EL, Eli village; GYH, Gaoyou Lake; HM, Haimen City; HQ, Huoqiu County; HZH, Hongze Lake; JL, Jiaoling County; LS, Lingshui County; PH, Pinghu City; PY, Puyang City; PYH, Poyang Lake; TH, Tai Lake; WSH, Weishan Lake; YN, Yunnan.

## 4 Discussion

*M. albus* lacks typical morphological characteristics, e.g., pectoral, pelvic, and caudal fins; therefore, molecular markers are effective to study its phylogeny and taxonomy (Nico et al., 2011; Cai et al., 2012). SNPs, as molecular markers, have been used widely to study the differentiation and genetic diversity of fish, e.g., in *Entosphenus Tridentatus* (Hess et al., 2013), *Salvelinus* spp. (Fraser et al., 2018), and *Misgurnus anguillicaudatus* (Yi et al., 2019). The results of SNP analysis usually reveal the most common polymorphisms in the genomes of most organisms (Brookes, 1999; Narum and Hess, 2011). In this study, 8941794 SNPs were detected in the 19 eel populations. The amount of genetic information obtained far exceeds that obtained using previous research methods, such as mitochondrial DNA and microsatellites (Cai et al., 2008; Li et al., 2013; Hu et al., 2016; Zhou et al., 2020). Therefore, SNP may be more reliable to judge the genetic diversity of eels.

Geographical isolation is one of the key factors leading to population differentiation. This study revealed that the LS population evolved from an independent ancestor. Moreover, there was extremely significant genetic differentiation between the LS population and the other populations. This might be because the town of Lingshui is located in the southeast of Hainan Island. This island is the second largest island in China and was separated from the mainland about 65 million years ago. The free migration, mating, and gene exchange between the LS population and mainland *M. albus* might be blocked, leading to the evolution of an almost independent LS population. The above situation was similar to that of the Ryukyuan population. Matsumoto’s study showed that Ryukyuan eels diverged from the Chinese-Japanese and Southeast Asian populations 5.7 million years ago to form separate genetic branches (Matsumoto et al., 2010). However, the genetic diversity of the LS population was extremely low (Pi < 0.01), which might weaken the ability of the population to withstand adversity and result in the LS *M. albus* being unable to survive in other habitats.

Jiaoling County and Eli village are located in the mountainous areas of southeast and southwest China. The *M. albus* collected from these areas had extremely significant genetic differentiation with the other populations. Moreover, the genetic diversity and inbreeding coefficient of the two populations were relatively low compared with those of other populations. These results might be related to the topography of the sample sites. Jiaoling County and Eli village are both surrounded by mountains, and have complex geographical features, including hills, mountains, basins, and valleys. These geographical factors would hinder gene exchange between local eels and outside populations. Therefore, similar to the LS population, the genetic characteristics of JL and EL *M. albus* were relatively primitive, and these populations were not suitable for long-distance rearing.

Poyang Lake is also located in Southwest China. The genetic structure analysis indicated that the *M. albus* of Poyang Lake (Jiangxi Province) evolved from one single ancestor. However, in another study, the JX population was shown to be a common descendant of both ancestors and to share the genetic information of clade A with other populations (Liang et al., 2016). Therefore, there may be genetic differentiation of *M. albus* in Jiangxi Province, which needs to be further verified. In addition, as the largest freshwater lake in China, the genetic diversity of eels in Poyang Lake is much lower than that in other lakes. Considering that Poyang Lake has been affected by overfishing in the recent years, it is not yet possible to decide whether the low genetic diversity was caused by historical legacy or human factors.

Previous study indicated that the lineages of coastal *M. albus* were immune from the expansion of inland populations (Cai et al., 2012). In this study, only DGS and PH populations were entirely descended from ancestor Ⅲ, despite the fact that the two populations are hundreds of kilometers apart. This may be because the DGS and PH *M. albus* originated from coastal areas, and thus might have derived some common genetic traits. Cai et al. found that three populations from regions adjacent to the East China Sea coastline can be divided into three distinct genetic lineages (Cai et al., 2012). This result was different from the present study. Further research is needed to determine whether the genetic differentiation of *M. albus* in coastal areas is more significant than that in inland areas. In addition, DGS *M. albus* showed significant genetic differentiation with the other populations. Moreover, the DGS population had relatively low genetic diversity. The DGS site is located in northeast China. There is no eel farming in northeast China (Heilongjiang, Jilin and Liaoning provinces). In fact, during our survey in Gushan county, we found that the local population had little enthusiasm for eels as food. Therefore, it can be deduced that the genetic diversity of *M. albus* was almost unaffected by artificial introduction.

The *M. albus* collected from the Yangtze River Delta, including Tai Lake, Hongze Lake, Gaoyou Lake, Haimen City, and Chongming Island, had the highest genetic diversity, with a Pi value ranging from 0.15 to 0.19. Moreover, these populations shared the genetic characteristics of both inland ancestor Ⅱ and coastal ancestor Ⅲ. This feature was similar to the results of other studies (Cai et al., 2012; Liang et al., 2016) This might be because the sampling sites were all large lakes and open waters, and the sample size of the eel population was large enough. Moreover, this area comprises the plains of the lower reaches of the Yangtze River. Thus, the genetic information of wild eels from sample sites near Yangtze River, e.g. Chengdu City, Dongting Lake, Poyang Lake, Chao Lake, and Huoqiu County, could converge on the Yangtze River Delta region through the Yangtze River system. In addition, we found high genetic diversity and a low inbreeding coefficient in the CM population. Moreover, the CM *M. albus* were closely related to the BYD, PY, and WSH populations. These results suggested that the phenomenon of artificial introduction was relatively frequent in the CM population. The Yangtze River Delta region is one of the largest distribution centers of aquatic products in China. Moreover, Chongming Island is connected with Shanghai city and Jiangsu Province, with convenient transport links, which has made this region the largest eel farming area in Shanghai. Thus, the genes of breeding value from different regions were more likely to converge into the eel population in Chongming Island through artificial introduction.

It should be noted that Huoqiu County and Chaohu Lake in Anhui province also belong to the Yangtze River Delta region; however, the populations from these two regions were significantly differentiated from TH, HZH, GYH, HM, and CM populations. In contrast, they were closely related to the CD, DTH, and YN populations in Southwest China. Moreover, we found that the CD, DTH, and CH populations evolved from a common ancestor, although Chaohu, Dongtinghu, and Chengdu, are located in the lower, middle, and upper reaches of the Yangtze River respectively. This might be because Anhui province lies at the intersection of the evolutionary lineages of different geographical groups (Cai et al., 2012; Hu et al., 2016). A previous study reported that eel populations in Anhui and Sichuan (including Chengdu City) provinces might originate from a single ancestor (Liang et al., 2016). However, another study suggested that genetically, swamp eels residing in the Sichuan Basin should probably be treated as a monogroup (Cai et al., 2008). Therefore, the genetic characteristics of *M. albus* residing in Anhui might be special, and more attention should be paid to artificial introduction.

In conclusion, there was significant genetic differentiation of *M. albus* in different regions of China. The genetic diversity of eels was the highest in Yangtze River Delta region, which is located in the plains of the lower reaches of the Yangtze River. However, the genetic diversity was the lowest in mountainous areas (JL and EL) and islands (LS). There was a close relationship between southwest populations (CD, DTH and YN) and the Anhui population. The coastal *M. albus* were distinctly differentiated from the inland populations. Therefore, it can be estimated that the genetic diversity of wild population was high and the germplasm resources were excellent. Further measures should be taken to protect these good wild resources and to convert them into artificial breeding parents, to make sustainable use of the wild germplasm resources of eel.

## Data Availability

The datasets presented in this study can be found in online repositories. The names of the repository/repositories and accession number(s) can be found below: https://www.ncbi.nlm.nih.gov/, SRX13783495-SRX13783631.
