# Supplementary material for: Asian Swamp eel Monopterus albus Population Structure and Genetic Diversity in China
Source: Front Genet. 2022 May 27;13:898958. doi: 10.3389/fgene.2022.898958 (PMC9198659; doi:10.3389/fgene.2022.898958)
Supplement: Supplementary file 1 [file Table1.docx]

**Table S1. Source of *Monopterus albus* samples.**

| **Locality** | **Code** | **Coordinates** | **Sample Size** |
| --- | --- | --- | --- |
| Baiyangdian Lake,  Hebei Prov. | BYD | 115° 57’E;  38° 57’N | 5 |
| Chengdu City,  Sichuan Prov. | CD | 104° 05’E;  30° 37’N | 10 |
| Chaohu Lake,  Anhui Prov. | CH | 117° 43’E;  31° 39’N | 8 |
| Chongming Island,  Shanghai City | CM | 121° 17’E;  31° 45’N | 5 |
| Gushan County,  Liaoning Prov. | DGS | 123° 38’E;  39° 56’N | 5 |
| Dongting Lake,  Hunan Prov. | DTH | 112° 57’E;  29° 25’N | 8 |
| Eli Village,  Guizhou Prov. | EL | 109° 09’E;  26° 35’N | 5 |
| Gaoyou Lake,  Jiangsu Prov. | GYH | 119° 24’E;  32° 58’N | 5 |
| Haimen City,  Jiangsu Prov. | HM | 121° 13’E;  31° 51’N | 4 |
| Huoqiu County,  Anhui Prov. | HQ | 116° 16’E;  32° 21’N | 10 |
| Hongze Lake,  Jiangsu Prov. | HZH | 118° 51’E;  33° 20’N | 10 |
| Jiaoling County,  Guangdong Prov. | JL | 116° 09’E;  24° 40’N | 9 |
| Lingshui County,  Hainan Prov. | LS | 110^。^04’E;  18^。^51’N | 6 |
| Pinghu City,  Zhejiang Prov. | PH | 110° 03’E;  18° 30’N | 10 |
| Poyang Lake,  Jiangxi Prov. | PYH | 116° 29’E;  29° 16’N | 10 |
| Puyang City,  Henan Prov. | PY | 115° 29’E;  35° 41’N | 10 |
| Tai Lake,  Jiangsu Prov. | TH | 120° 23’E;  31° 27’N | 5 |
| Weishan Lake,  Shandong Prov. | WSH | 117° 18’E;  34° 39’N | 5 |
| Dali City,  Yunnan Prov. | YN | 100° 13’E;  25° 48’N | 7 |
| Total |  |  | 137 |
